# Case report: Initial atypical skeletal symptoms and dental anomalies as first signs of Gardner syndrome: the importance of genetic analysis in the early diagnosis

**DOI:** 10.3389/pore.2024.1611768

**Published:** 2024-05-14

**Authors:** Gréta Antal, Anna Zsigmond, Ágnes Till, Eniko Orsi, Ildiko Szanto, Gergely Büki, László Kereskai, Zsuzsanna Herbert, Kinga Hadzsiev, Judit Bene

**Affiliations:** ^1^ Department of Dentistry, Oral and Maxillofacial Surgery, Clinical Center, Medical School, University of Pécs, Pécs, Hungary; ^2^ Department of Medical Genetics, Clinical Center, Medical School, University of Pécs, Pécs, Hungary; ^3^ Department of Pathology, Clinical Center, Medical School, University of Pécs, Pécs, Hungary; ^4^ Department of Medical Imaging, Clinical Center, Medical School, University of Pécs, Pécs, Hungary

**Keywords:** Gardner syndrome, *APC* gene, osteoma, dental abnormalities, WES

## Abstract

**Background:** Gardner syndrome is a rare genetic cancer predisposition disorder characterized by intestinal polyposis, multiple osteomas, and soft and hard tissue tumors. Dental anomalies are present in approximately 30%–70% of patients with Gardner syndrome and can be discovered during routine dental examinations. However, sometimes the diagnosis is challenging due to the high clinical variability and incomplete clinical picture. Herein, we report a family with various dental and bone anomalies, in which the definitive diagnosis was established with the help of a comprehensive genetic analysis based on state-of-the-art next-generation sequencing technology.

**Case presentation:** A 17-year-old female index patient presented with dental (caries, impacted, retained and anteriorly located teeth) and atypical bone anomalies not resembling Gardner syndrome. She was first referred to our Genetic Counselling Unit at the age of 11 due to an atypical bone abnormality identified by a panoramic X-ray. Tooth 3.6 was surgically removed and the histopathology report revealed a Paget’s disease-like bone metabolic disorder with mixed osteoblastic and osteoclastic activity of the mandible. A small lumbar subcutaneous tumor was discovered by physical examination. Ultrasound examination of the tumor raised the possibility of a soft tissue propagation of chondromatosis. Her sister, 2 years younger at the age of 14, had some benign tumors (multiple exostoses, odontomas, epidermoid cysts) and impacted teeth. Their mother had also skeletal symptoms. Her lower teeth did not develop, the 9th-10th ribs were fused, and she complained of intermittent jaw pain. A cranial CT scan showed fibrous dysplasia on the cranial bones. Whole exome sequencing identified a heterozygous pathogenic nonsense mutation (c.4700C>G; p.Ser1567*) in the *APC* gene in the index patient’s DNA. Targeted sequencing revealed the same variant in the DNA of the other affected family members (the sister and the mother).

**Conclusion:** Early diagnosis of this rare, genetically determined syndrome is very important, because of the potentially high malignant transformation of intestinal polyps. Dentists should be familiar with the typical maxillofacial features of this disorder, to be able to refer patients to genetic counseling. Dental anomalies often precede the intestinal polyposis and facilitate the early diagnosis, thereby increasing the patients’ chances of survival. Genetic analysis may be necessary in patients with atypical phenotypic signs.

## Introduction

Gardner syndrome (GS) is a rare autosomal dominant inherited disorder characterized by a triad of gastrointestinal polyposis, multiple osteomas and hard and soft tissue tumors [1]. It is caused by a germline variation in the adenomatous polyposis coli (*APC*) tumor suppressor gene on chromosome 5q21-22 [2]. This syndrome is a clinical variant of familial adenomatous polyposis (FAP), affecting approximately 10% of FAP patients with an incidence of 1:8300–16000 births [3, 4]. The *APC* germline mutation shows almost complete penetrance with respect to the intestinal phenotype, but extraintestinal manifestations are not present in all affected patients [5]. By the fourth decade of life, the frequently asymptomatic colon polyps will progress to malignant transformation in almost all patients without treatment [6, 7]; therefore, early diagnosis and appropriate patient management are crucial for patient prognosis and survival.

Approximately 30%–70% of patients with GS present with dental abnormalities such as osteomas of the jaw, supernumerary teeth, odontomas, hypodontia, impacted or unerupted teeth, and abnormal tooth morphology [8]. These orofacial features often precede the intestinal polyposis and may serve as an early marker of this syndrome, so the dentist may play a key role in the early establishment of the diagnosis [9]. However, in a certain number of cases, the diagnosis is challenging due to the diverse clinical manifestations. In these cases, molecular genetic analysis allows for an earlier diagnosis.

Here we report a family with three affected members with variable clinical manifestations. The proband had atypical bone anomalies, the diagnosis of which was established with the help of whole exome sequencing. Our cases emphasize the important role of genetic analysis in patients with dental features, especially in cases where dental and oral anomalies occur together with extra-oral manifestations.

## Case description

**FIGURE 1:**
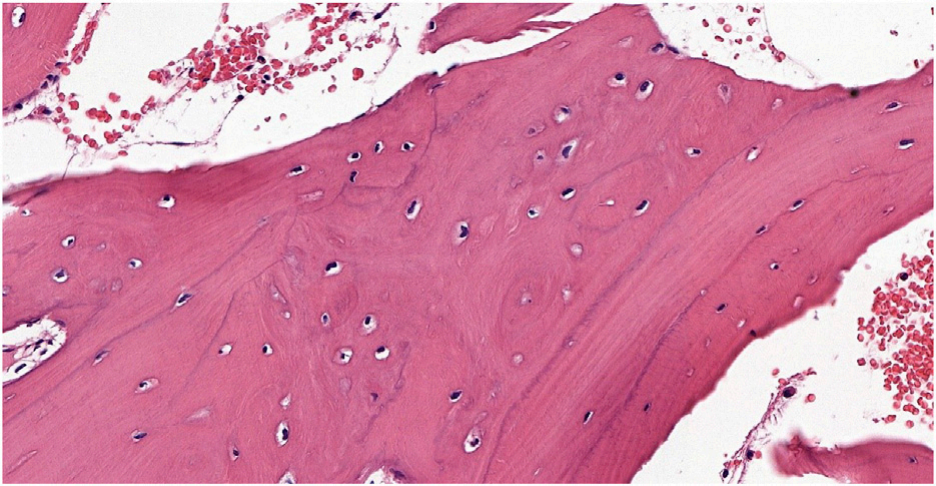
The biopsy from the proband shows a bone sample with widened trabeculae. Osteoblastic activity and irregular bone formation are seen as blue cement lines. The cement lines represent changes in the direction of new bone formation and are not pathognomonic of a specific process by themselves; they are best seen in Paget’s disease but may be visible in other disorders involving rebuilding of the bone structure (Hematoxylin-Eosin stain 9.9x).

**FIGURE 2:**
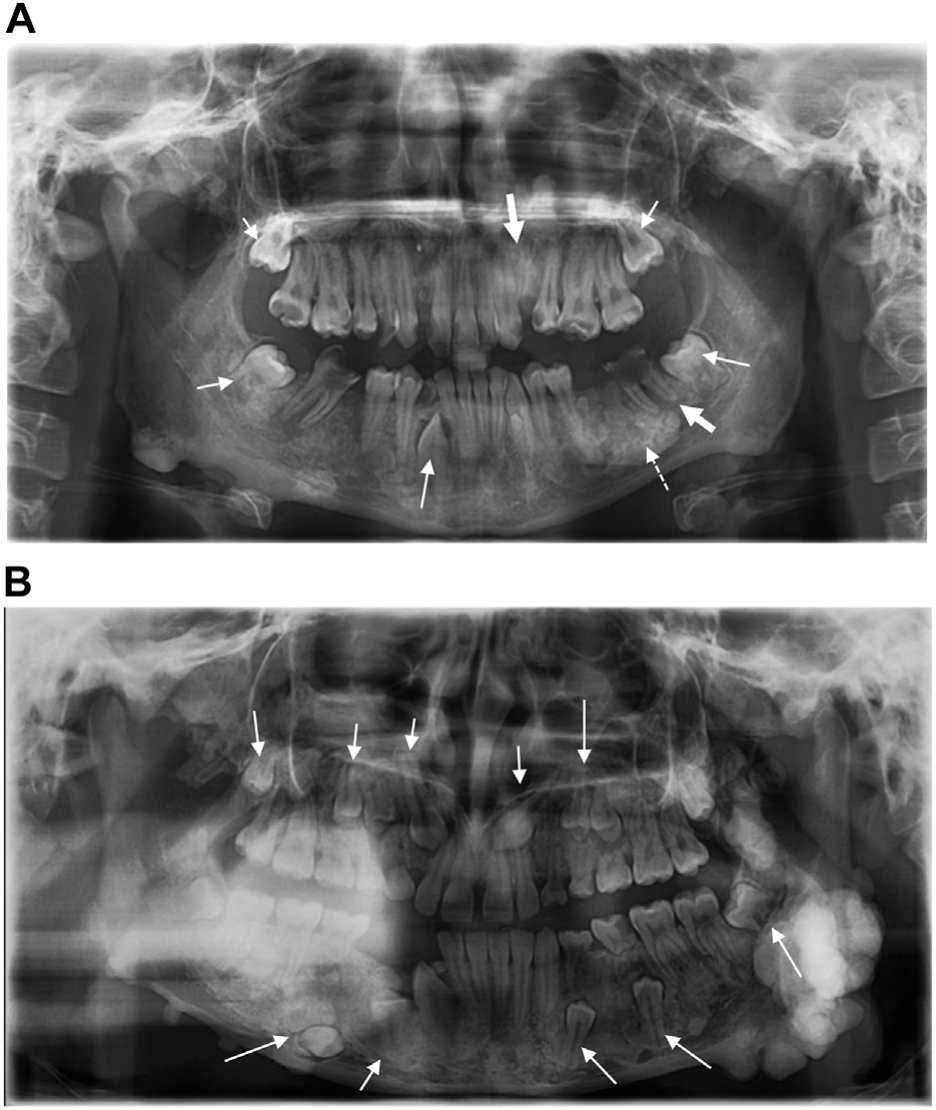
**(A)** Panoramic radiograph of the proband shows impacted teeth (small arrows), radix relicta (large arrows), and the dotted arrow indicates the location of the specimen taken for histopathological examination. Dental caries are present in almost all teeth **(B)** Panoramic radiograph of the proband’s sister shows multiple impacted teeth.

An 11-year-old female patient was referred to the Genetic Counseling Unit of our Department by the Oral Surgery Clinic, University of Pécs. During the dental examination bone abnormalities were revealed by a panoramic X-ray. Tooth 3.6, which caused uncertain complaints was surgically removed and a sample was taken from the abnormal bone area of the mandible. Histopathological examination revealed a Paget’s disease-like bone metabolic disorder with mixed osteoblastic and osteoclastic activity (Figure 1). A small lumbar subcutaneous tumor was discovered by physical examination of the patient. Ultrasound examination of the tumor raised the possibility of soft tissue propagation of chondromatosis. Lumbar spine X-rays showed spina bifida occulta of the SI segment without structural abnormalities of the bone. These findings suggested early-onset Paget’s disease type 2 as the underlying cause of the patient’s symptoms. Comprehensive genetic testing was not available at that time. Five years later, a dental examination of the patient at the age of 16 revealed poor oral hygiene, with small to large caries lesions on almost all teeth. The 4.3 tooth was retained. Wisdom teeth 3.8 and 4.8 have a mesioangular impaction (Figure 2A). Due to the suspicion of Paget’s disease, a new sample was taken under local anesthesia during the removal of tooth 3.7. Cortical and spongiosal bone samples were taken from the mandibular corpus corresponding to the root of the previously removed tooth 3.6. Histopathological examination revealed a tissue structure reminiscent of Paget’s disease. A next-generation sequencing (NGS) skeletal dysplasia panel test comprising 252 genes was performed, which did not identify a pathological variant underlying the symptoms. Subsequently, an extension of the genetic analysis to whole exome sequencing (WES) was initiated.

**FIGURE 3:**
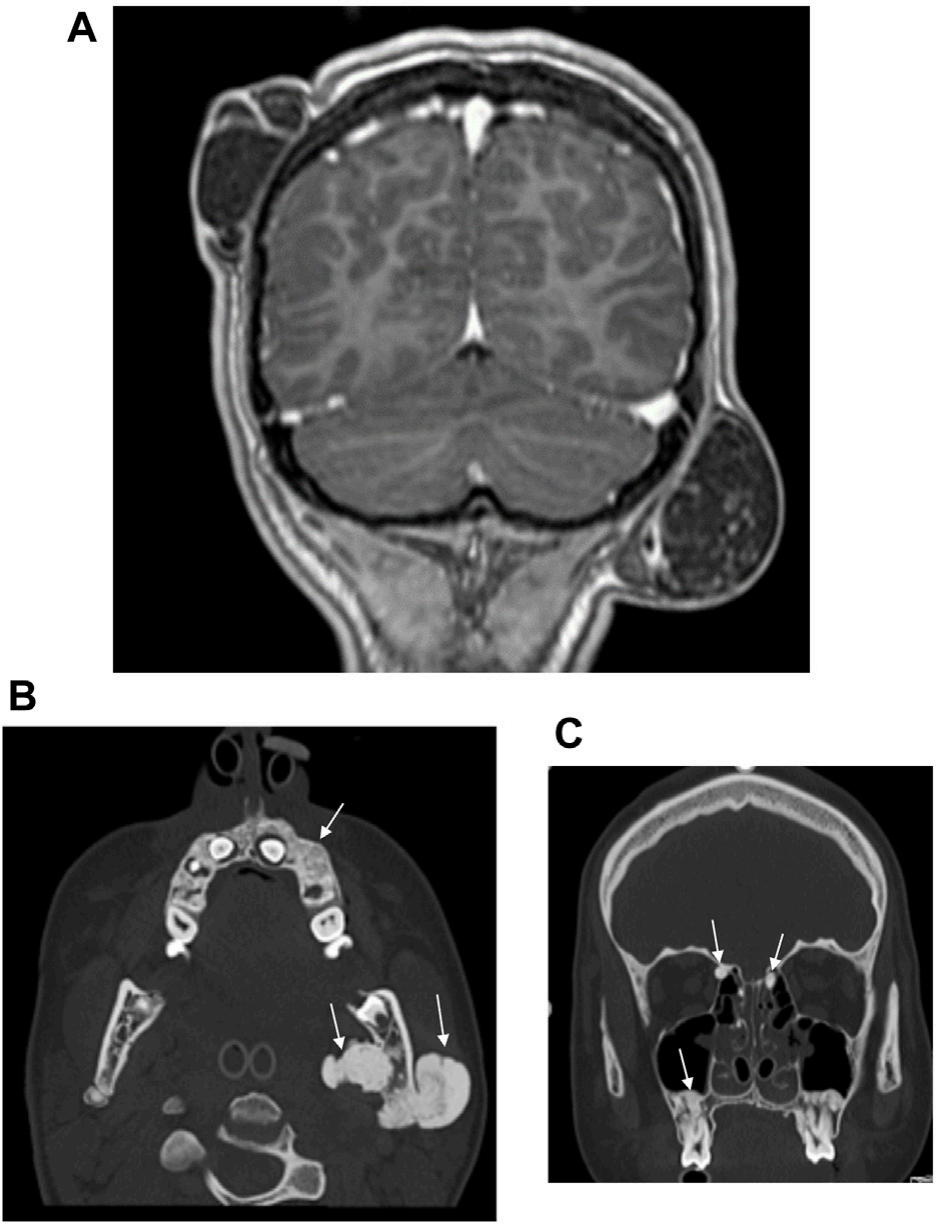
**(A)** Postcontrast coronal T1W MP RAGE MRI reveals right parietal and left occipital dermoid cysts. **(B)** CT examination of the skull shows osteomas of the left mandibular ramus and alveolar process of the maxilla. **(C)** CT examination of the skull reveals multiple osteomas of the ethmoid cells and alveolar process of the maxilla.

At the same time, the proband’s 14-year-old sister presented to our Genetic Counselling Unit with skeletal and soft tissue manifestations. Her medical history included the following: at the age of 11 years, two tumors had been removed from the subcutaneous connective tissue of the head, one from the right parietal region and one from the left occipital region. The histological examination confirmed the existence of dermoid cysts. At the age of 14 years, a dermoid cyst was removed, again from the left occipital region. At the same time, a painful tumor appeared on her left mandible. One month later, an acute cranial computed tomography (CT) scan was performed because of a convulsion lasting more than 40 min. Multiple exostoses affecting the cranial bones were described and the mandible was distinctly sclerotic. Electroencephalography (EEG) did not confirm epileptic activity. Three months later uncertain neurological symptoms developed, with motor aphasia noted in addition to a left temporal headache. A sleep deprivation EEG also showed no organic signs or paroxysmal dysfunction. Finally, a complicated migraine was considered to be the underlying cause of the headache and motor aphasia. To clarify the diagnosis, cranial magnetic resonance (MR) imaging was performed, but no intracerebral abnormality was found. The multiple bone lesions depicted may be consistent with osteomas when evaluated together with the earlier CT scan images (Figure 3). Based on the radiological findings GS was suspected and the patient was admitted to oncology care. In addition to the mandibular tumor, her detailed physical examination revealed minor mobile subcutaneous lesions on the lateral side of the right knee and above the proximal interphalangeal joint of the right index finger. The patient also underwent a maxillofacial examination due to multiple mandibular exostoses and an age-inappropriate eruption status of the teeth. A tumor in the mandibular area of the left angulus-ramus mandibularis was seen on extraoral examination. Exostoses of approximately 0.5 cm were palpable basally on the right side. Intraoral examination revealed an eruption status of the teeth, canines and premolars were in rentention (Figure 2B). The deciduous teeth had caries, while the remaining teeth were intact. A cone beam computed tomography (CBCT) scan showed 18–20 mm of cortical consistency in exostoses around the mandibular ramus, not compressing the cancellous bone. The mother also presented with skeletal symptoms. Teeth agenesis was observed in her mandible, the 9th-10th ribs were fused, and she complained of intermittent jaw pain. A cranial CT scan showed multiple osteosclerotic lesions on the cranial bones. Gastroenterological and oncological investigations have not been performed yet.

Whole exome sequencing (WES) identified a pathogenic nonsense mutation (c.4700C>G; p.Ser1567*) in the *APC* gene [NM_000038.6] in heterozygous form in the proband’s DNA sample. This variation was detected in both the mother’s and the younger sibling’s DNA samples by targeted testing. Based on the phenotype of the patients and their genetic test results, the diagnosis of GS was clearly confirmed.

## Discussion and conclusion

GS is a rare genetic cancer predisposition disorder characterized by pleiotropy and genetic heterogeneity. The syndrome is linked to a germline mutation in the Adenomatous Polyposis Coli (*APC*) gene encoding the 300 kDa Anaphase-promoting complex protein. *APC* is a tumor suppressor gene involved in cell proliferation, migration, adhesion, cytoskeletal stabilization, chromosome segregation, apoptosis, and neuronal differentiation [10–12]. The majority of the patients (approximately 75%) have a positive family history, but spontaneous mutations occur in 25%–33% of the individuals [9].

GS is a form of familial adenomatous polyposis that affects multiple systems; the clinical features can be classified into intestinal and extra-intestinal manifestations. Intestinal manifestations comprise colonic adenomatous polyps (tubular, villous, tubulovillous), small intestinal adenomatous polyps and periampullary carcinomas. Extra-intestinal features include congenital hypertrophy of the retinal pigment epithelium (CHRPE), osteomas, fibromas, lipomas, epidermal and sebaceous cysts and desmoid tumors [13–16]. Dental anomalies associated with GS manifest in 30%–70% of patients. They may comprise supernumerary teeth, compound odontomas, hypodontia, impacted or unerupted teeth, hypercementosis [10, 17], dentigerous cysts, and caries [8]. Symptoms usually manifest by the end of the second decade of life, but they can occur anytime from 2 months to 70 years. The maxillofacial symptoms of the syndrome may manifest several years before the intestinal polyposis; therefore, dental professionals should be aware of the significance of the syndrome as a pre-cancerous condition. Panoramic radiography can be a useful tool in the early detection of GS. The elements of this syndrome, such as osteoma, odontoma, supernumerary teeth, and impacted teeth, can be identified on routine radiological examination [18, 19]. However, panoramic radiography is of limited value in the evaluation, localization, and extension of the tumor mass, considering the superimposition of the bony structures along with the fact that it is a two-dimensional image [20].

The clinical diagnosis of GS is often challenging due to the variable expressivity of the extra-intestinal manifestations [4, 21]. Osteomas are the hallmark of GS and their presence is required to establish the diagnosis. They are found mainly in the mandible, but osteomas may occur in the skull, paranasal sinuses, and long bones [22]. In the mandible, the classic location of osteomas is the mandibular angle and inferior surface [18]. Our index patient presented only dental (impacted and retained teeth) and bone anomalies without osteomas; therefore, the symptoms did not imply the clinical diagnosis of GS. Finally, the diagnosis was established with the help of comprehensive genetic analysis based on state-of-the-art next-generation sequencing technology. WES revealed a pathogenic mutation in the *APC* gene. Interestingly, the proband’s younger sister showed the classic clinical features of GS: multiple osteomas, odontomas, epidermoid cysts, and impacted teeth. In this case, the clinical diagnosis was confirmed by targeted mutation analysis. The mother of the siblings also carried the disease-causing mutation. She has dental and skeletal manifestations, but these do not fulfill the GS phenotype. None of our patients had intestinal symptoms at the time of diagnosis; however, the mother refused colonoscopy for her children and herself, and as a result we have no information about intestinal polyps in our patients. Moreover, our patients did not have CHRPE. The maternal grandfather had a laryngeal tumor and the grandmother had a cardiac enlargement. Because the maternal grandparents had died several years before, genetic testing for them was not available. Based on this information we believe that the mutation was developed *de novo* in the mother. During post-test genetic counseling, the family was informed about the prognosis, increased risk of tumor occurrence, mode of inheritance, risk of recurrence and prevention options. Based on the National Comprehensive Cancer Network 2020 recommendation, we made a suggestion to the affected family members about the importance of screening tests [23].

Many rare diseases have dental manifestations [24]. According to the London Dysmorphology Database, in 2011, dental-oromaxillofacial anomalies were present in the clinical pictures of approximately 900 out of 5000 genetic syndromes [25]. Dental and oral anomalies together with extraoral symptoms can facilitate the early diagnosis of rare genetic syndromes. In a certain number of cases, dental malformations may be the most evident manifestations, and therefore the first to be diagnosed, while other symptoms affecting different organs may be discovered later [25]. In certain rare disorders, such as GS, which is a cancer predisposition syndrome, early diagnosis is of great importance. Thanks to state-of-the-art genetic testing methods, such as NGS-based targeted sequencing or whole exome sequencing, molecular genetic diagnosis is possible in the majority of these syndromes.

Dentists should be aware of the existence of rare genetic syndromes, such as GS, and know how to clinically recognize, treat and manage the dento-oro-craniofacial anomalies that patients may have. Moreover, it is of great importance to refer these patients for genetic counseling. Therefore it would be advisable to include the discussion of GS in undergraduate and postgraduate training materials and exams. The highly variable clinical features require multidisciplinary care for patients with GS [26]. Although dental anomalies may be an early clinical indicator of this cancer predisposition syndrome, genetic/gastroenterological evaluation is much more important for patients than treatment of dental abnormalities or the removal of exostoses.

In summary, we reported the case of a family with GS. The index patient had multiple symptoms and WES analysis provided an early diagnosis. Our cases emphasize the importance of genetic counseling and genetic testing in patients with dento-oro-craniofacial anomalies. Several rare genetic diseases have dental or orofacial manifestations that can be the first symptoms of serious, life-threatening manifestations such as in GS. Therefore, it is important for dentists to be aware of these symptoms and to refer these patients for genetic counseling. Modern genetic testing can quickly and effectively confirm the diagnosis allowing a screening plan and an early intervention to be initiated, which can greatly increase the patients’ chances of survival.

## Data Availability

The raw data supporting the conclusion of this article will be made available by the authors, without undue reservation.

## Abbreviations

APC, adenomatous polyposis coli; CBCT, cone beam computed tomography; CHRPE, congenital hypertrophy of retinal pigment epithelium; CT, computed tomography; EEG, electroencephalography; FAP, familial adenomatous polyposis; GS, Gardner syndrome; MR, magnetic resonance; NGS, next-generation sequencing; WES, whole exome sequencing.
